# Upregulation of Cardiac IL-10 and Downregulation of IFN-*γ* in Balb/c IL-4^−/−^ in Acute Chagasic Myocarditis due to Colombian Strain of *Trypanosoma cruzi*

**DOI:** 10.1155/2018/3421897

**Published:** 2018-11-28

**Authors:** Marcos Vinicius da Silva, Vera Lúcia de Almeida, Wendyson Duarte de Oliveira, Natália Carasek Matos Cascudo, Pollyana Guimarães de Oliveira, Crislaine Aparecida da Silva, Aline Cristina Souza da Silva, Maria Luíza Gonçalves dos Reis Monteiro, Rosana Rosa Miranda Correa, Milton Adriano Pelli Oliveira, Ruy de Sousa Lino-Júnior, Mara Rúbia Nunes Celes, Juliana Reis Machado

**Affiliations:** ^1^Department of Parasitology, Institute of Natural and Biological Sciences, Federal University of Triângulo Mineiro, 38025-180 Uberaba, MG, Brazil; ^2^Institute of Tropical Pathology and Public Health of Federal University of Goiás, Federal University of Goiás, 74605-050 Goiânia, Brazil; ^3^General Pathology, Institute of Natural and Biological Sciences, Federal University of Triângulo Mineiro, 38025-180 Uberaba, MG, Brazil

## Abstract

Inflammatory response in Chagas disease is related to parasite and host factors. However, immune system regulation has not been fully elucidated. Thus, this study is aimed at evaluating IL-4 influence on acute phase of *Trypanosoma cruzi* experimental infection through dosage of cytokine levels in cardiac homogenate of infected Balb/c WT and Balb/c IL-4^−/−^ as well as its histopathological repercussions. For such purpose, mice were divided into two groups: an infected group with 100 forms of the Colombian strain and an uninfected group. After 21 days of infection, animals were euthanized and the blood, spleen, and heart were collected. The spleen was used to culture splenic cells in 48 h. Subsequently, cytokines TNF-*α*, IL-12p70, IL-10, IFN-*γ*, and IL-17 were measured in the blood, culture supernatant, and heart apex by ELISA. The base of the heart was used for histopathological analysis. From these analysis, infected Balb/c IL-4^−/−^ mice showed milder inflammatory infiltrate compared to Balb/c WT, but without changes in nest density and collagen deposition. IL-4 absence culminated in lower cardiac tissue IFN-*γ* production, although it did not affect TNF-*α* expression in situ. It also decreased TNF-*α* systemic production and increased IL-10, both systemically and *in situ*. In addition, IL-4 absence did not influence IL-17 expression. Splenocytes of IL-4-deficient mice produced higher amounts of IFN-*γ*, TNF-*α*, and IL-17 and lower amounts of IL-10. Thus, IL-4 absence in acute phase of experimental infection with *T. cruzi* Colombian strain reduces myocarditis due to lower IFN-*γ* production and greater IL-10 production *in situ* and this pattern is not influenced by splenocyte general repertoire.

## 1. Introduction

Chagas disease, also known as American trypanosomiasis, is a chronic systemic infectious disease caused by the protozoan *Trypanosoma cruzi (T. cruzi)*, an important public health problem in American continent. According to the World Health Organization (WHO), the disease affects around 7.5 million people worldwide, the majority in Latin America [1, 2].

Murine experimental model reproduces human Chagas' disease infections, and genetic background has influences both, triggering a series of reactions that evolve from an acute symptomatic phase to chronic phase [3]. Some variables interfere in Chagas' disease pathogenicity such as the infecting strain and the mouse lineage. Balb/c strain mice are known to be susceptible to *T. cruzi* infection, present a high parasitemia and the vast majority succumb early to acute infection [4]. Colombian strain has been used in several studies with the purpose of evaluating cardiac function due to its evident myotropism [5]. Prominent myocardial lesions with intense inflammatory process, evolution in parasitemia with peak between 21 and 25 days of postinfection, distinct myotropism with involvement of the skeletal musculature resulting in extensive lesions in the skeletal muscle fibers, electrocardiographic alterations, and resistance to chemotherapeutics such as benznidazole and nifurtimox are characteristics of this strain. [6, 7].

Knockout model is widely used in studies that evaluate direct or indirect implication of certain cytokines in resistance to *T. cruzi* infection. IL-10 KO mice infected with *T. cruzi* Y strain had lower serum and tissue parasite load and higher IFN-gamma and nitric oxide production by spleen cells than wild-type mice [8]. Furthermore, IL-10 KO mice infected with the Tulahuen strain, in addition to lower serum parasite load, also showed higher levels of serum TNF-*α*, IL-12, and IFN-gamma compared with infected IL-10^+^/^+^ mice [9]. Knockout models for other molecules as B2-microglobulin [10], CD4 or CD8 [11], gamma delta T cells [12], and Fas ligand [13] were also used in studies on Chagas disease.

In acute phase, Chagas disease has variable clinical presentations, from asymptomatic to symptomatic and even fatal in some cases. Parasite proliferation and dissemination by blood or lymphatic vessels characterized this phase, with a parasitemia peak that regresses in some days and tends to be undetectable in chronic phase. Acute phase dissemination affects several cellular types, especially cardiac muscle fibers, with amastigotes nest formation [14], myocarditis, and subsequent collagen deposition between cardiac fibers, causing progressive functional damage. Excessive immune system activation with proinflammatory cytokine production of Th1-type IL-12, TNF-*α*, and IFN-*γ* characterizes this phase. On the other hand, cytokines such as IL-10 and TGF-*β* are activated to modulate immune response with consequent reduction of tissue damage [15, 16].

IL-4 is described as an anti-inflammatory cytokine, prototypic of Th2 profile, and related to susceptibility to protozoa infection, such as cutaneous leishmaniasis [17, 18], although even in models of polarized clinical manifestations, its relation with Th1 response still raises major controversies [19]. IL-4 reduces IFN-*γ* production [20] controlling Th1 response and preventing excessive tissue inflammation [21]. IL-4 knockout animals tend to have better response to infection by intracellular parasites, and when infected by *T. cruzi*, they increase Th1-type immune response, resulting in decreased parasitemia and mortality and increased cardiac inflammatory reaction, although these effects are observed in late stages of acute or in chronic phase [21, 22]. On the basis of *in vitro* testing, two contrasting roles for IL-4 have been described: enhancement of intracellular *T. cruzi* destruction by macrophages [23] and inhibition of IFN-*γ*-mediated trypanocidal activity [24].

Our group had already pointed out that infected and reinfected animals with Colombian strain present a modulation of immune response leading to higher production of proinflammatory cytokines, such as IFN-*γ* and TNF-*α*, resulting in marked myocarditis and lower survival rate [25]. As Balb/c mice are usually good IL-4 producers [26] and due to IL-4 relevance in modulating response against infectious and parasitic agents, including chagasic cardiomyopathy, IL-4^−/−^ knockout animals were used to analyze if this cytokine has any influence on immune response modulation in mice infected with *T. cruzi* Colombian strain, which has marked myotropism in acute phase. We demonstrate that in early stages of acute phase, IL-4^−/−^ knockout animals presented milder myocarditis with lower IFN-*γ* production and higher in situ IL-10 production, despite Th1 cells increase in splenocyte general repertoire. Our results open perspectives of IL-4 role in chagasic myocarditis initial events in a more complex frame than the dichotomy Th1/Th2.

## 2. Material and Methods

### 2.1. Animals and Infection

We evaluated male animals of Balb/c WT and Balb/c IL-4^−/−^ lineages infected with *T. cruzi* Colombian strain (*n* = 8) and an uninfected group (*n* = 6), both aged 10 weeks and weighing 20-25 g. Infections were performed using trypomastigote forms obtained at peak of parasitemia from previously infected Swiss mice (Waynforth and Flecknell, 1992). Briefly, Swiss mice were euthanized and peripheral blood was collected by cardiac puncture. Five microliters of blood was used to quantify parasite number in a hematocytometer, counted in fifty microscopic fields at a final magnification of ×400. Inoculum was adjusted to 1000 trypomastigotes/ml. One hundred (100) trypomastigote forms were subcutaneously inoculated. Animals were euthanized on the 21st day of infection for acute phase. Next, necropsy was performed and the heart was collected through a ventral incision in thoracic cavity. This research was approved by CEUA/UFG.

### 2.2. Histological Analysis

To evaluate inflammatory infiltrate, slides of cardiac tissue (ventricle) stained with hematoxylin and eosin (HE) were used. Qualitative analysis classified infiltrate as predominantly mononuclear (macrophages and lymphocytes) or polymorphonuclear (neutrophils and eosinophils), according to the cellular type observed in more than 50% of the infiltrate. Semiquantitative analysis classified the inflammatory infiltrate in mild (involvement < 25% of the tissue), moderate (25%–50% of the tissue), or severe (involvement > 50% of the tissue) [25].

Tissue parasitism was quantified under light microscopy by the number of myocardium *T. cruzi* nests using three slides with serial slices. All infected animals had nests.

For collagen quantification, we used the heart sections stained with sirius red. Slides were analyzed in polarized light microscope at a final magnification of ×400 with a semiautomatic interactive image analyzer system, ImageJ® (National Institutes of Health, Bethesda, EUA).

### 2.3. Immunological Analysis

#### 2.3.1. Cardiac Tissue Homogenate Preparation

The heart tissue sections were immersed in PBS solution containing complete protease inhibitor (Sigma, St. Louis, MO, USA) and Nonidet-P40. After that, they were submitted to tissue homogenizer. The homogenate obtained was centrifuged at 14,000 ×g for 10 minutes, and the supernatant was maintained for quantification of cytokines and total proteins [25].

#### 2.3.2. Culture of Splenocytes

Mouse splenocytes were collected, maintained in RPMI 1640 medium (GE Healthcare, Uppsala, Sweden) and macerated for cell individualization. These suspended cells were washed three times by centrifugation at 400 ×g for 15 min at 8°C in RPMI 1640. Then, they were counted in a Neubauer chamber and resuspended to 2 × 10^6^ cells/ml in RPMI 1640 medium with addition of 50 mM Hepes (Gibco, Grand Island, NY, USA), 5% of inactivated fetal bovine serum (Gibco, USA), 2 mM L-glutamine (Gibco, USA), 0.05 mM 2*β*-mercaptoethanol (Gibco, USA), and 40 *μ*g/ml gentamicin (Neoqumica, Anápolis, GO, BR). Then, 2 × 10^6^ cells were incubated without stimulus or with 10 *μ*g/ml of concanavalin A in 24-well culture plates (BD Pharmingen, San Diego, CA, USA). Cultures were kept in a moist incubator with 5% CO_2_ at 37°C for 48 hours. Supernatants were collected and maintained at −70°C until analysis [25].

#### 2.3.3. TNF-*α*, IL-12p70, IL-10, IFN-*γ*, and IL-17 Quantification

Cytokine quantification was performed on cardiac homogenate, spleen culture, and serum cells by ELISA (enzyme-linked immunosorbent assay), using antibody pairs from BD commercial kit (Biosciences, USA) and following manufacturer's instructions. Reaction was developed using 3,3′,5,5′-tetramethylbenzidine (TMB) peroxidase substrate and read at 450 nm. For cardiac homogenate, results were normalized to total protein concentration, determined by Bradford assay (Bio-Rad, Hercules, CA, USA) of each heart and expressed as picogram of cytokine per gram of tissue (pg/g of tissue).

### 2.4. Statistical Analyses

The GraphPad Prism 6.0 software (GraphPad Software, USA) was used. Student's *t*-test was used for analysis between two groups with normal distribution.

Qualitative variables were expressed as percentage and associations were analyzed using the chi-square (*χ*^2^) test. Results were considered statistically significant when *p* < 0.05.

## 3. Results

### 3.1. Balb/c IL-4^−/−^ Mice Infected with *T. cruzi* Strain Presented Less Intense Inflammatory Infiltrate in Acute Phase of Infection

**Figure 1:**
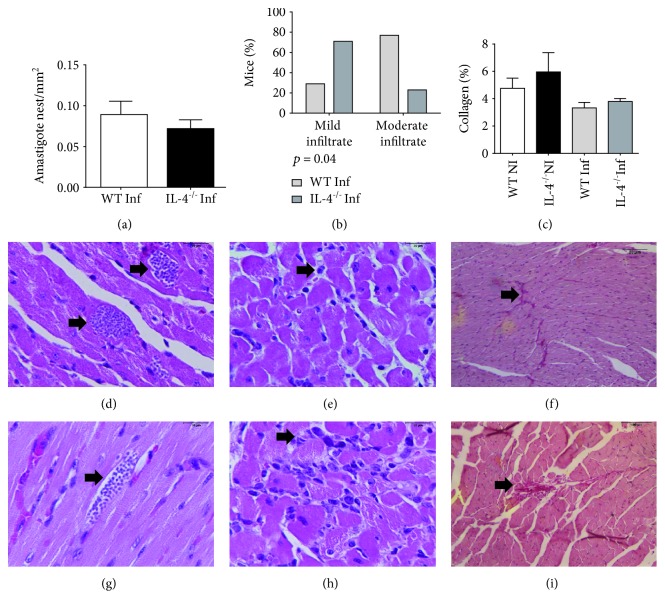
Morphological analysis of the heart of Balb/c WT and Balb/c IL-4^−/−^ mice infected and not infected with the Colombian strain of *T. cruzi* in the acute phase of experimental Chagas' disease. (a) Density of amastigote nests in cardiac tissue of infected WT and IL-4^−/−^ mice. Bars represent the mean, and vertical lines represent the standard error. (b) Intensity of cardiac inflammatory infiltrate in infected WT and IL-4^−/−^ mice (*p* = 0.04, chi-square test). (c) Percentage of collagen fibers in cardiac tissue of infected and uninfected WT and IL-4^−/−^ mice. Bars represent the mean, and vertical lines represent the standard error. Histological sections of the WT and IL-4^−/−^-infected mice heart. (d, g) HE-stained amastigote nests. (e) Mild inflammatory infiltrate. (h) Moderated inflammatory infiltrate. (f, i) Collagen fibers stained red by sirius red.

Inflammatory cardiac infiltrate in Balb/c WT and Balb/c IL-4^−/−^ mice infected with Colombian strain in acute phase of experimental Chagas disease was analyzed. IL-4 absence had an impact on inflammatory infiltrate reduction, as Balb/c IL-4^−/−^ had predominantly moderate inflammatory infiltrate (*p* = 0.04, chi-square test, Figure 1). Regarding quantification of heart fibrosis and amastigotes nests, no difference was observed in group comparison (Figure 1).

### 3.2. Absence of IL-4 Culminates in Lower IFN-*γ* Cardiac Tissue Production with Similar Expression of TNF-*α*

Once infection with *T. cruzi* Colombian strain leads to lower inflammatory infiltrate in IL-4 knockout animals, we evaluated immune response quality in situ through cardiac homogenate and systemically through serum dosages. As expected, wild-type animal infection triggered increased IFN-*γ* production in cardiac tissue (noninfected WT vs. infected WT, *p* = 0.02, *t* = 2.611). However, in IL-4 absence, IFN-*γ* expression was significantly reduced, either basal (NI WT vs. NI IL-4^−/−^, *p* = 0.009, *t* = 3.315) or after infection with Colombian strain (Inf WT vs. Inf IL-4^−/−^, *p* = 0.001, *t* = 4.43). No statistically significant difference was observed in IFN-*γ* serum levels (data not shown).

However, despite significant IFN-*γ* reduction in cardiac tissue, TNF-*α* expression was similar between WT and IL-4^−/−^, both in uninfected mice (NI WT vs. NI IL-4^−/−^, *p* > 0.17) and after infection (Inf WT vs. Inf IL-4^−/−^, *p* > 0.5). In both groups of animals, infection with Colombian strain induced a significant increase in TNF-*α* (NI WT vs. Inf WT, *p* = 0.02, *t* = 2.61 and NI IL-4^−/−^ vs. Inf IL-4^−/−^, *p* = 0.002, *t* = 4.74). Interestingly, TNF-*α* systemic levels were significantly elevated in WT-infected animals compared to IL-4^−/−^ animals (*p* = 0.01, *t* = 3.08).

**Figure 2:**
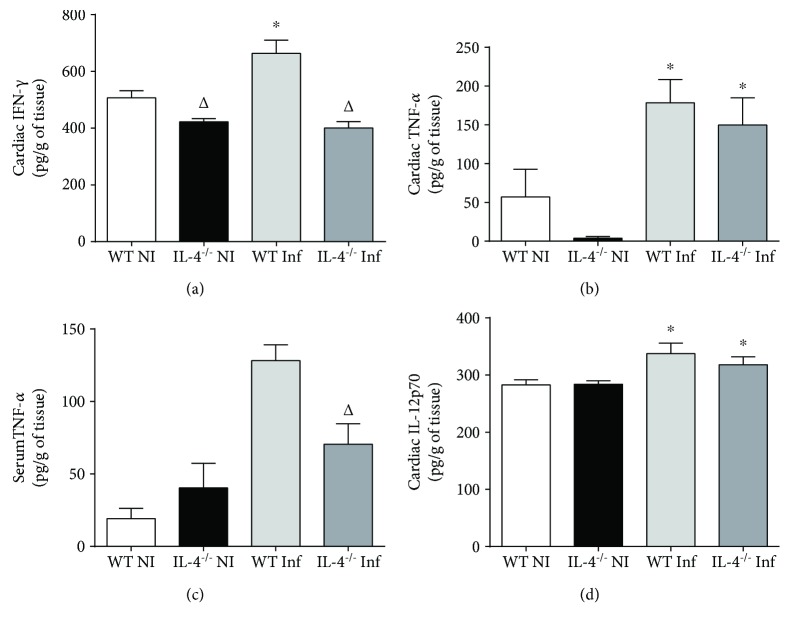
Expression of proinflammatory cytokines in cardiac tissue and serum of Balb/c WT and Balb/c IL-4^−/−^ mice infected and not infected with the Colombian strain of *T. cruzi* in the acute phase of experimental Chagas' disease. (a) Tissue IFN-*γ* production (pg/g). (b) Tissue TNF-*α* production (pg/g). (c) Serum levels of TNF-*α* (pg/ml). (d) Tissue IL12p70 production (pg/g). Student's *t*-test. Bars represent the mean, and vertical lines represent the standard error. ^∗^Significant differences between infected versus uninfected animals. ^Δ^Significant differences between the WT versus IL-4^−/−^ group.

To investigate if nonproduction of IFN-*γ* was due to nonexpression of Th1-inducing cytokines, we evaluated in situ expression of IL-12p70. In both groups, infection induced significantly increased IL-12p70 expression in cardiac tissue (NI WT vs. Inf WT, *p* = 0.04, *t* = 2.32 and NI IL-4^−/−^ vs. Inf IL-4^−/−^, *p* = 0.04, *t* = 2.39), but with no difference between WT and IL-4^−/−^, *in situ* or systemically (*p* > 0.05, Figure 2).

### 3.3. Lower Inflammation and Lower IFN-*γ* Production in Cardiac Tissue of IL-4^−/−^ Animals Infected with *T. cruzi* Colombian Strain Are due to Increased IL-10 Production

Once we observed Balb/c mice with IL-4 absence and infected with *T. cruzi* cardiotropic strain had a significant IFN-*γ* expression reduction in cardiac tissue, and that this reduction was not due to a decrease in innate immunity cytokine production related to Th1 cell differentiation or function, such as TNF-*α* and IL-12p70, we evaluated a possible differential production of IL-10, classically implicated in anti-inflammatory mechanisms.

**Figure 3:**
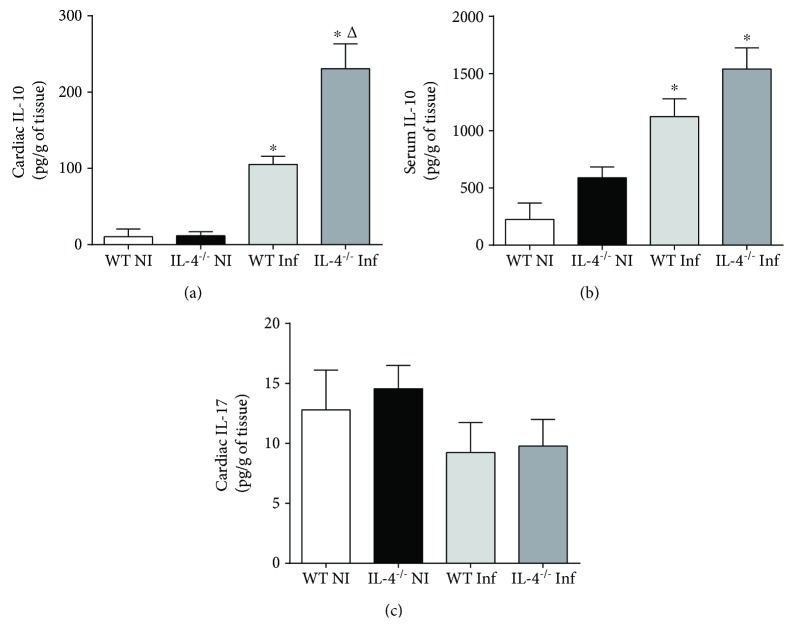
Cytokine expression with regulatory profile in cardiac tissue and serum of Balb/c WT and Balb/c IL-4^−/−^ mice infected and not infected with the *T. cruzi* Colombian strain in the acute phase of experimental Chagas' disease. (a) Production of IL-10 in cardiac tissue (pg/g) and (b) serum levels of IL-10 (pg/ml). (c) Production of IL-17 in cardiac tissue (pg/g). Student's *t*-test. Bars represent the mean, and vertical lines represent the standard error. ^∗^Significant differences between infected versus uninfected animals. ^Δ^Significant differences between the WT versus IL-4^−/−^ group.

Our results indicate that *T. cruzi* Colombian strain infection induced significant IL-10 production, both in WT mice (NI WT vs. Inf WT, *p* = 0.007, *t* = 5.29) and Balb/c IL-4^−/−^ (NI IL-4^−/−^ vs. Inf IL-4^−/−^, *p* = 0.001, *t* = 4.93). Furthermore, in IL-4 absence, significantly higher IL-10 levels were observed in cardiac tissue following infection (Inf WT vs. Inf IL-4^−/−^, *p* = 0.004, *t* = 3.42). A significant increase in serum IL-10 between WT and IL-4 animals^−/−^ (*p* > 0.05) was not observed, although both had higher systemic levels after infection (NI WT vs. Inf WT, *p* = 0.002, *t* = 4.25 and NI IL-4^−/−^ vs. Inf IL-4^−/−^, *p* = 0.001, *t* = 4.89) (Figure 3).

Finally, considering that IL-4 absence could imply alterations of other non-Th1 inflammatory profiles, we assessed Th17 profile through IL-17 in situ expression. No statistical differences were observed in IL-17 cytokine expression between groups, which shows that nonactivation of Th2 profile by IL-4 absence did not lead to Th17 profile activation in a compensatory fashion (Figure 3).

### 3.4. Cytokine Pattern in Cardiac Tissue Is Not due to Difference in Splenocyte General Repertoire

**Figure 4:**
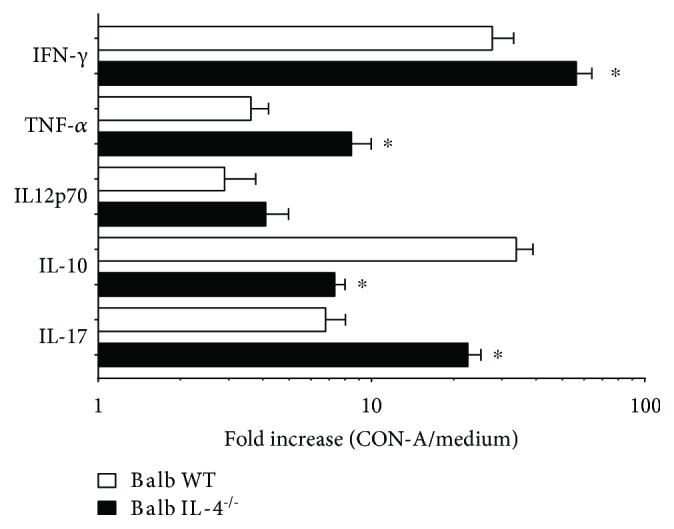
Cytokine production in splenocytes of WT Balb/c and IL-4^−/−^ Balb/c mice infected with Colombian strain of *T. cruzi* stimulated with 10 *μ*g/ml of concanavalin A in the acute phase of experimental Chagas' disease. Fold change between nonstimulated versus CON-A stimulated splenocytes. Student's *t*-test. Bars represent the mean, and vertical lines represent the standard error. ^∗^Significant differences between infected versus uninfected animals.

**Figure 5:**
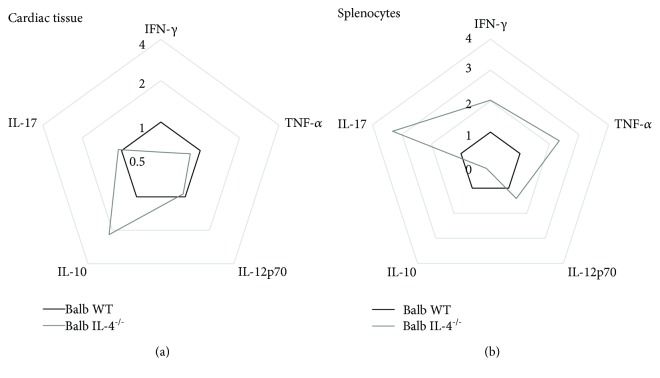
Radar plot representation of cytokine profile in cardiac tissue and splenocytes. The lines highlight the fold change in cytokine production in IL-4^−/−^ Balb/c (gray line) in relation to WT Balb/c mice (black line). Data were obtained by calculating the ratio between the mean concentrations of each cytokine in the IL-4^−/−^ Balb/c-infected group and WT Balb/c-infected mice.

To assess if observed differences in cardiac tissue cytokine expression of IL-4^−/−^ animals were due to a global difference in lymphocyte repertoire, we stimulated splenocytes with 10 *μ*g/ml of concanavalin A for 48 h and calculated cytokine production (expressed in fold increment related to nonstimulated splenocytes). We demonstrate here that IL-4^−/−^ mouse splenocytes produce significantly increased amounts of IFN-*γ* (Inf WT vs. Inf IL-4^−/−^, *p* = 0.02, *t* = 2.79), TNF-*α* (Inf WT vs. Inf IL-4^−/−^, *p* = 0.009, *t* = 3.39), IL-17 (Inf WT vs. Inf IL-4^−/−^, *p* = 0.006, *t* = 5.47), and lower IL-10 production (Inf WT vs. Inf IL-4^−/−^, *p* = 0.007, *t* = 5.76) (Figure 4). This result shows a clear difference between repertoire generated by *T. cruzi* Colombian strain infection and cytokines expressed at the infection site, more clearly demonstrated by radar plots (Figure 5). These results were calculated using the mean expression of each cytokine in cardiac tissue homogenate or CON-A-stimulated splenocytes from Balb/c IL-4^−/−^-infected mice and expressed as fold change relative to Balb/c WT-infected mice.

## 4. Discussion

This study is aimed at evaluating IL-4 role in immune response modulation of mice infected with *T. cruzi* Colombian strain in acute phase of infection. Our results point out the relationship between IL-4 and Th1 cells, classically described as antagonistic. In acute myocarditis triggered by cardiotropic strain of *T. cruzi*, IL-4 absence implies a general polarization for Th1 in the spleen, but in cardiac tissue, inflammatory balance is significantly regulated by an increase in IL-10, triggering a lower inflammatory infiltrate.

The profile of cytokines released during *T. cruzi* infection may be associated with a protective or disease susceptible profile. Th1-type response is characterized by IL-2 and IFN-*γ* secretion, leading to activation of macrophages and cell-mediated response, whereas in Th2 response, synthesis of IL-4, IL-5, and IL-10 is observed, culminating in relevant humoral response. Balance between these cytokines is fundamental to determine which response has to be developed [27, 28].

In our study, IL-4 absence at 21 days of infection did not determine a change in amastigotes nest density in cardiac tissue, using 100 forms of Colombian strain of *T. cruzi*, a reference strain for chagasic myocarditis studies due to its high heart tropism in Balb/c mice [29, 30]. These results conflict with other studies that demonstrated the potentiating IL-4 effect on infection, since IL-4^−/−^ mice would better control tissue parasitism [21, 31]. However, here and in both studies, nest density was small, even though several serial cuts were evaluated. Another study using greater inoculum with Y strain found no difference in parasite load as did our study [32]. Taken together, all these studies show in the early events of *T. cruzi* infection that intense myocarditis has no direct relation with the amount of amastigote nests that will persist in chronic phase.

In the present study, Balb/c IL-4^−/−^-infected mice had milder inflammatory infiltration compared to WT Balb/c. No significant differences were observed in intensity of diffuse and focal inflammation in cardiac tissue compared IL-4 KO and WT animals [31]; however, this work evaluated late acute phase in 30 days of infection, where parasitemia is reduced and transition to chronic infection begins, while we evaluate the moment when Colombian strain presents its parasitemia peak, that is, greater parasitic circulation [25].

Fibrosis is a fundamental substrate of Chagas' heart disease and progression to heart failure [33]. However, there is no consensus on the exact moment process takes place. In face of a continuous inflammatory process, it is believed that extracellular matrix increases collagen production evolving to fibrosis, especially secondary to tissue damage. In the present study, difference in collagen deposition was not found, possibly due to the time infection was evaluated, when myocarditis is prominent and fibrosis is not yet fully installed. In chronic phase, it is well established that induction of inflammatory response and death of cardiac fibers contribute to continuous deposition of collagen and consequently fibrosis [1, 34].

IL-4 absence culminated in lower cardiac tissue IFN-*γ* production; this fact justifies decrease of in situ infiltrate. However, IFN-*γ* expression is extremely relevant for immunity against intracellular pathogens, including *T. cruzi* [35]. We believe systemic production of IFN-*γ* suppressed local decrease of this cytokine, since its levels were well expressed in splenocytes culture in the IL-4^−/−^ group. However, increased IFN-*γ* and nitric oxide production was demonstrated in mice infected with *T. cruzi* Tulahuen strain with IL-4 suppression, showing that IL-4 absence is related to a greater proinflammatory state activation in *T. cruzi* infection [36].

IL-4 absence did not alter TNF-*α in situ* production, despite IFN-*γ* local reduction. TNF-*α* is especially synthesized by macrophages, T lymphocytes, and NK cells, with a great diversity of functions including recruitment and activation of macrophages, which stimulate nitric oxide production and intracellular destruction of protozoan, actively participating in the initial proinflammatory response of the disease [37, 38]. Other studies have shown that increased TNF-*α* is also associated with increased cardiac damage [39, 40], although treatment with TNF-*α* inhibitors, such as etanercept, in chronic phase, aggravates chagasic myocarditis [41], demonstrating that its presence in cardiac tissue has more complex repercussions than conceptual simplification increase is equal to damage. It is important to emphasize that splenocyte production capacity was positively impacted by IL-4 absence, suggesting cardiac tissue-specific control mechanisms.

Among infected animals, there was no difference in IL-12p70 cytokine expression in IL-4 absence. It is known that this cytokine is a fundamental mediator of innate immune response, secreted by mononuclear phagocytes and dendritic cells, and important in stimulating IFN-*γ* production by NK cells and T lymphocytes [42, 43]. It can be inferred that, in this study, at the beginning of infectious process, the ability to produce IL-12p70 contributes to a Th1 response profile influenced by pathogen itself.

In fact, animals in the present study had no histological evidence of cardiac involvement. We believe this is due to immune response control represented in our study by in situ increase in IL-10 and decrease in IFN-*γ*. Our data also point out that immune response local regulation associated with a good repertoire of systemic Th1 immune response may have been sufficient to maintain the effectiveness of the response to *T. cruzi* infection. These data become more consistent, since we observed increased serum and *in situ* IL-10 with reduced systemic repertoire production, as demonstrated in stimulated culture.

IL-4 absence did not influence IL-17 expression *in situ*; however, the repertoire in the ex vivo culture increased. IL-17 plays an important role in resolution of *T. cruzi* protozoan infection, and this cytokine is associated with protective and nonpathogenic responses [44–46]. In chagasic patients with heart disease, greater serum IL-17 levels were related to improvement of organ function; therefore, it has protective effect [47]. A study in IL-17^−/−^ animals demonstrated that this cytokine actively participates in inflammatory response in initial phase of disease and its absence during *T. cruzi* infection results in a reduction in recruitment of defense cells, which favors parasitemia [48]. However, researches are still recent and limited on true role of this cytokine in Chagas' disease.

The relationship between IL-4 functions and Th1 cell differentiation is classically described as antagonistic and widely reported in several models of T lymphocyte differentiation [49–52]. However, we demonstrated in acute myocarditis triggered by *T. cruzi* cardiotropic strain that IL-4 absence implies a repertorial polarization for Th1, but in cardiac tissue, inflammatory balance is strongly regulated by an increase in IL-10, triggering a lower inflammatory infiltrate. Our results open perspectives of IL-4 role in initial events of chagasic myocarditis in a more complex frame than the dichotomy Th1/Th2.

## Data Availability

The datasets generated during and/or analyzed during the current study are available from the corresponding author on reasonable request.

## Abbreviations

CON-A: Concanavalin A
ELISA: Enzyme-linked immunosorbent assay
IFN-*γ*: Interferon gamma
IL-4: Interleukin 4
IL-10: Interleukin 10
IL-17: Interleukin 17
IL-12p70: Interleukin 12p70
KO: Knockout for the gene
*T. cruzi*: *Trypanosoma cruzi*
TNF-*α*: Tumor necrosis factor-alpha
TMB: 3,3′,5,5′-Tetramethylbenzidine
WT: Wild type.
